# Digital expression profiling of the compartmentalized translatome of Purkinje neurons

**DOI:** 10.1101/gr.164095.113

**Published:** 2014-08

**Authors:** Anton Kratz, Pascal Beguin, Megumi Kaneko, Takahiko Chimura, Ana Maria Suzuki, Atsuko Matsunaga, Sachi Kato, Nicolas Bertin, Timo Lassmann, Réjan Vigot, Piero Carninci, Charles Plessy, Thomas Launey

**Affiliations:** 1 RIKEN Center for Life Science Technologies, Division of Genomic Technologies, Yokohama, Kanagawa, 230-0045 Japan;; 2 RIKEN Brain Science Institute, Launey Research Unit, Wako, Saitama, 351-0198 Japan

## Abstract

Underlying the complexity of the mammalian brain is its network of neuronal connections, but also the molecular networks of signaling pathways, protein interactions, and regulated gene expression within each individual neuron. The diversity and complexity of the spatially intermingled neurons pose a serious challenge to the identification and quantification of single neuron components. To address this challenge, we present a novel approach for the study of the ribosome-associated transcriptome—the translatome—from selected subcellular domains of specific neurons, and apply it to the Purkinje cells (PCs) in the rat cerebellum. We combined microdissection, translating ribosome affinity purification (TRAP) in nontransgenic animals, and quantitative nanoCAGE sequencing to obtain a snapshot of RNAs bound to cytoplasmic or rough endoplasmic reticulum (rER)–associated ribosomes in the PC and its dendrites. This allowed us to discover novel markers of PCs, to determine structural aspects of genes, to find hitherto uncharacterized transcripts, and to quantify biophysically relevant genes of membrane proteins controlling ion homeostasis and neuronal electrical activities.

The emergence of the system approach to the study of neuron function came from the realization that no protein or process can function in isolation but is often embedded in a network of regulating interactions. While often detailed, no study of signaling networks can claim to be exhaustive, for lack of a “parts list” of all the components, and also because of the limited precision regarding the concentration of the ones known to be involved. For many neurons the presence of an extended dendritic arbor provides spatial constraints and additional complexity since remote or semi-isolated compartments may create local and transient conditions. The consequence is that biophysical in silico models remain inefficient for predicting the alteration of electrical activities under disease or exposure to drugs. Thus, rather than yielding a unique model, fitting of available experimental data results in sets of equally good (and equally bad) nonunique models (Achard and De Schutter 2006) that are also incomplete. For instance, for Purkinje cells (PC), the most complete and realistic models only include less than 20 distinct proteins (Miyasho et al. 2001; Korogod and Tyc-Dumont 2009).

The cataloging of building parts is further complicated by its dynamic nature, with protein concentration being modified through transcriptional and post-transcriptional regulation, as well as local destruction or synthesis of components. These modifications are nevertheless functionally important because protein synthesis in general and especially local synthesis in dendrites are required for synapse maturation and plasticity (Martin and Ephrussi 2009; Liu-Yesucevitz et al. 2011). This has motivated several recent efforts for large-scale transcriptome analysis both for single neuronal-type translatome (Doyle et al. 2008; Heiman et al. 2008; Knight et al. 2012) and specifically for the dendrite/neuropil compartments (Poon et al. 2006; Zhong et al. 2006; Cajigas et al. 2012).

Part of the PC transcriptome has been previously explored using purification strategies based on differential expression between the wild type and a PC-devoid mutant (Rong et al. 2004), laser microdissection (Friedrich et al. 2012), or neuron-type–specific capture of ribosome (Doyle et al. 2008; Heiman et al. 2008). This last approach, termed translating ribosome affinity purification (TRAP), is especially attractive as it targets RNAs bound to ribosomes (the “translatome”) rather than the full population of transcribed RNAs. Currently, however, this approach and the related RiboTag strategy (Sanz et al. 2009) have been used to establish all-or-none gene expression by specific cell type, while the quantitative estimation of RNA translation has not been exploited. Quantification of translating mRNA is expected to be a better proxy measurement of protein synthesis (Schwanhäusser et al. 2011) than the total mRNA level, which has long been recognized as a poor predictor of protein abundance (Gygi et al. 1999). The use of both TRAP and RiboTag is practically limited to mice since these strategies require transgenic animals engineered to express a modified ribosomal protein (RPL10 for TRAP and RPL22 for RiboTag). In addition, detailed analysis of the generated data sets identified the need for extensive processing and filtering to remove contaminants and nonlinearities (Dougherty et al. 2010).

Here, we combined several of the approaches presented above to identify RNAs present in the PC and in its dendrites, associated with either the cytoplasmic or endoplasmic reticulum–bound fraction of the ribosomes (Fig. 1A). To detect and quantify RNAs in the PC’s translatome, we used the CAGE method (cap analysis of gene expression), which predominantly detects the transcription start sites (TSS) and measures their abundance by quantitatively sequencing the 5′ end of cDNAs from capped mRNAs (Shiraki et al. 2003), independently of transcript length or presence of a polyadenylated tail. In our study, since the available RNA was limited in quantity and the genome of the chosen model animal, *Rattus norvegicus* is not annotated as extensively as for mouse or human, we chose the high-sensitivity paired-end nanoCAGE/CAGEscan implementation of CAGE (Plessy et al. 2010) to identify TSS independently of existing annotation.

**Figure 1.**
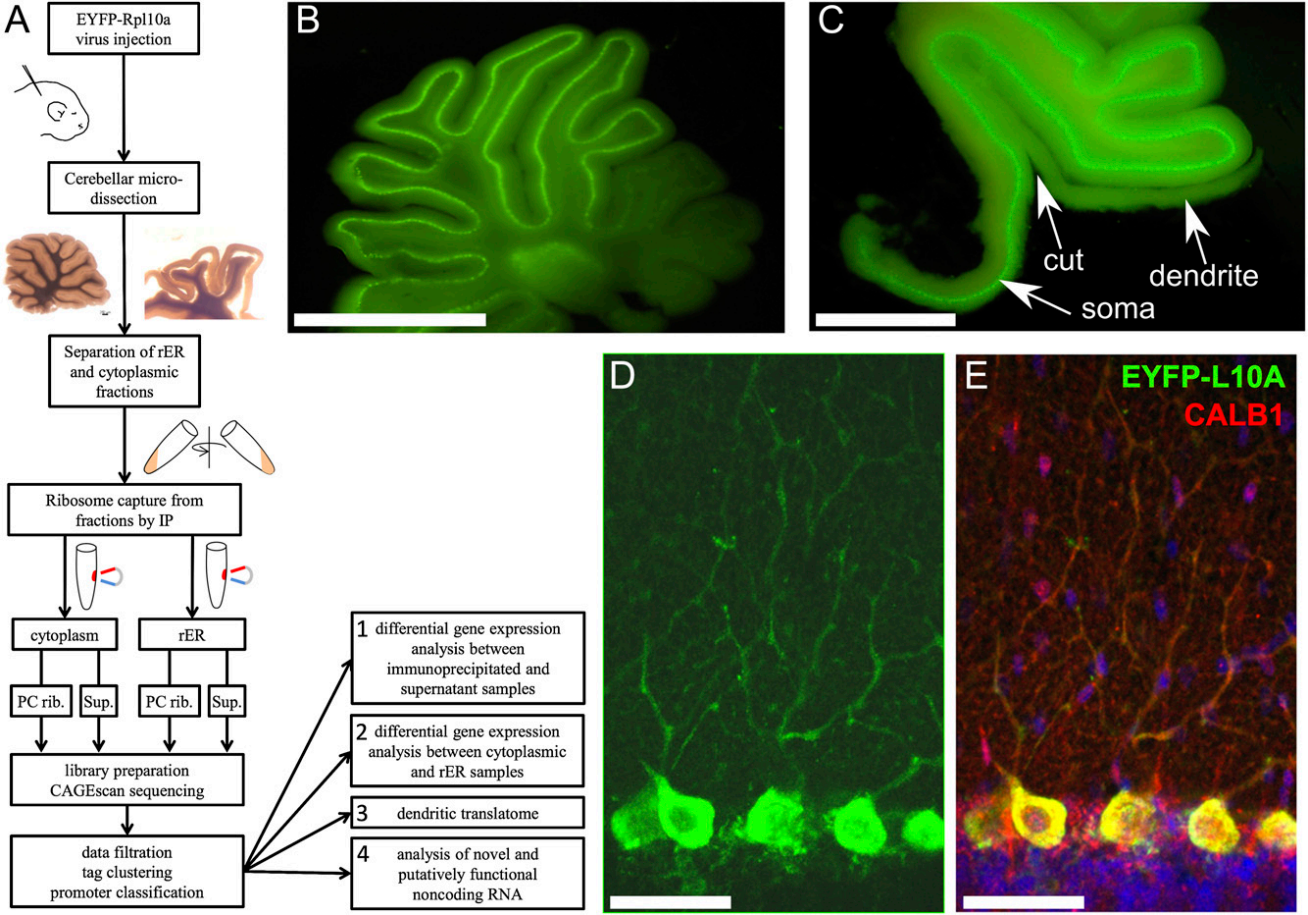
(*A*) General layout of the study. (*B*) Micrograph of a live cerebellar slice (rat) showing expression of EYFP-RPL10A in the PCs and occasionally in deep-cerebellar nuclei. (*C*) Microdissection of the molecular layer to separate PC somata from dendrites. Scale bar, 1.76 mm. Note that most of the granular layer under the PC layer is also removed to reduce contamination. Scale bar, 2.42 mm. (*D*) Immunofluorescence detection of EYFP-L10A (anti-GFP) after fixation, showing intense staining of PC somata and weaker signal in dendrites. (*E*) Single optical section of combined staining for EYFP-L10A (green), calbindin (red), and DAPI (blue). Scale bar for *D* and *E*, 58 μm.

## Results

### Ribosome capture followed by sequencing reveals the translatome of a specific neuronal cell type

To specifically target an EYFP-RPL10A ribosome-capture construct to PCs without the generation of transgenic animals, we used a mosaic AAV virus (AAV2/2-8) combining capsid proteins from the AAV2 and AAV8 isotypes to maximize transduction in PCs (Broekman 2006). Preliminary comparisons showed that AAV1 and AAV2 are less efficient (for both) and less specific (for AAV1) to transduce PCs, confirming the observation of Broekman (2006). Intracerebellar injection of AAV2/2-8 at P4 resulted in intense expression of the EYFP-RPL10A construct in up to eight lobules of the vermis (Fig. 1B), variable spread to lateral hemisphere (Supplemental Fig. S1), and expression restricted to PCs, both in mice (data not shown) and rat (Fig. 1B–E). The specificity achieved through capsid selection allowed us to use a strong CAG promoter, without interfering with PC’s endogenous promoters (Fig. 1D,E). The EYFP variant (Miyawaki et al. 1999) chosen here is brighter than the EGFP used by Heiman et al. (2008), allowing microdissection of the cerebellar cortex under fluorescence illumination (Fig. 1C). The 40S ribosome proteins RPL29 or RPL36, previously found to be enriched in PCs (Sato et al. 2008), were also examined as an alternative to RPL10A, as the ribosome anchor for the TRAP construct (Doyle et al. 2008; Heiman et al. 2008). We did not observe any evidence for differential distribution or abundance for any of the tested proteins relative to RPL10A (data not shown). Since RPL10A itself was found to be present in Purkinje dendrites (see below), we used it as the ribosome-targeting component of the probe.

By microdissection of live cerebellar vermal slices, nine pools comprising the Purkinje and molecular layers of 57 ± 7 lobules, restricted to lobules IV to IX (692 in total) (Supplemental Table S1) were prepared. Only the cerebellar vermis was studied since this is most relevant as comparison with electrophysiological studies of cerebellar plasticity and since PCs in this region show homogeneous gene expression while the cerebellar lateral hemispheres show region-specific gene expression (Oberdick et al. 1993). To provide functional context to our characterization of the PC translatome, each sample was separated into a cytoplasmic and a rough endoplasmic reticulum (rER)–bound fraction. The transcripts from the two fractions showed different size profiles (Supplemental Fig. S2A–D), with longer RNAs in the cytoplasm compared with the rER, and yielded on average 260 ± 51 and 188 ± 35 ng of total RNA, respectively. In addition, to analyze the dendritic translatome, we pooled the tissue from 80 lobules, microdissected it to isolate the upper two-thirds of the molecular layer containing the dendritic trees of the PCs, yielding 7.79 ng of RNA (Supplemental Fig. S2E,F). For two pools, we sequenced the supernatants remaining after the immunopurification in order to assess the quality and specificity of ribosome capture.

To analyze the 5′ transcriptome of these submicrogram samples, we prepared a total of 24 nanoCAGE libraries, using random reverse-transcription primers in order to detect transcripts regardless of the presence or absence of a poly(A) tail. After quantitative sequencing of the libraries, we could align 73,544,526 paired-end reads to the rat genome. PCR noise was canceled by collapsing identically aligned pairs for a final number of 27,740,924 CAGE tags. A detailed count of the tags in each library before and after alignment is available in Supplemental Table S1.

CAGEscan libraries have more PCR duplicates than RNA-seq libraries because the first read in CAGE is anchored to the 5′ end of cDNAs, while RNA-seq reads represent randomly fragmented cDNAs. Our libraries had 1.2 ± 0.8 million reads after removing PCR duplicates (see Supplemental Table S1). Random subsampling of our data confirmed that diminishing returns would be expected when increasing sequencing depth, both in terms of number of significant differences in statistical comparisons and the fold change of these differences (Supplemental Figs. S3, S4).

### Clustering and machine learning identify a subset of high-confidence promoters

We grouped the whole single-nucleotide resolution CAGE signal into clusters representing functional units. In this article, we use the term TSS as in the Sequence Ontology (Eilbeck et al. 2005) term SO:0000315, “the first base where RNA polymerase begins to synthesize the RNA transcript,” and the term “cluster” for groups of neighboring 5′ ends of CAGE tags.

**Figure 2.**
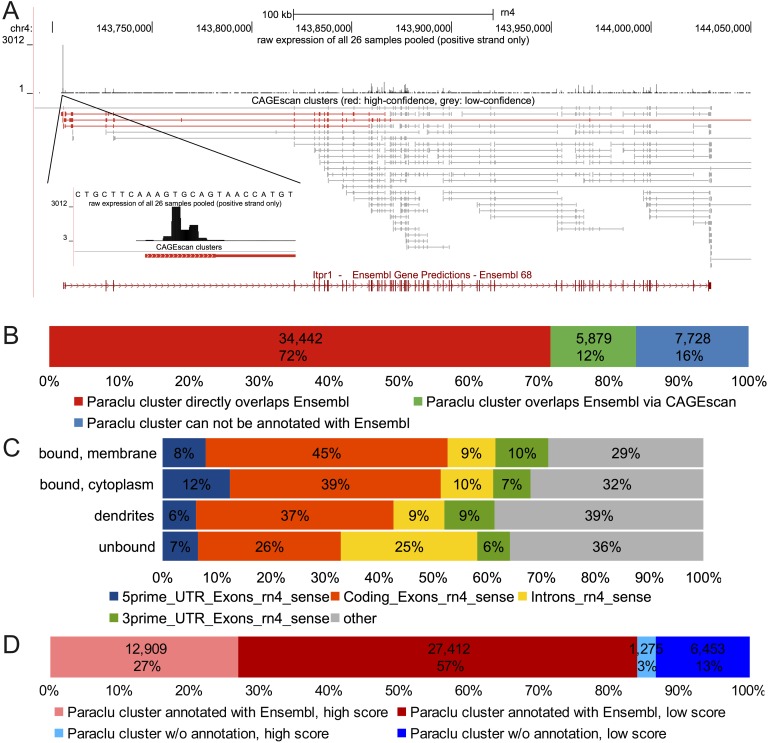
(*A*) Genomic representation of the PC marker *Itpr1*. *Below* the chromosomal coordinates are shown the following: (*top*) quantitative representation of CAGE tag abundance on the positive strand and (*bottom*) Paraclu followed by CAGEscan clustering groups tag into clusters (here color-coded according to the cluster’s classification score). (*B*) Paraclu clusters annotated with an Ensembl gene by direct overlap or via CAGEscan. (*C*) Percentages of the first 5′ nucleotide sequences that fall into 5′ untranslated regions, exons, introns, and 3′ untranslated regions of Ensembl genes (downloaded March 28, 2012). (*D*) Promoter classification.

By using the Paraclu peak calling algorithm (Frith et al. 2008) and setting a maximum length of 100 bp, we obtained 48,049 clusters. Each Paraclu cluster was then used as a seed position to assemble a CAGEscan cluster, consisting of the original Paraclu cluster, followed by the collated 3′-mates of the pairs originating from it (Fig. 2A; Supplemental File S1). The sequencing depth was sufficient to make CAGEscan clusters resemble the intron–exon structure of the assembled transcripts. Clusters were then matched with annotated Ensembl transcripts (Flicek et al. 2013), overlapping in sense. Of the 48,049 clusters, 40,321 could be annotated with 9550 different genes, while 7728 clusters could not be annotated and may correspond to novel promoters of known transcripts or to promoters of entirely novel transcripts. Among the annotated clusters, 5879 would not have an annotation if not using CAGEscan (Fig. 2B).

The nanoCAGE protocol enriches for capped RNA and therefore the 5′ mates should map to the beginning of the annotated transcripts. Nevertheless, a considerable amount of tags map within coding exons and intronic regions (Fig. 2C), with some clusters located deep within known transcripts. Six thousand sixty-five genes are represented by more than one cluster, and while some of these could represent alternative downstream TSSs, some of this background noise may stem from capped processed transcripts (Fejes-Toth et al. 2009) or present features that render them easy to capture by template switching. To separate true signal from potential background, we reasoned that if a large number of basal promoters shared some sequence features, it would be possible to separate clusters representing promoters from the other clusters. We therefore constructed a machine learning classifier and trained it with known promoters. The classifier models the distributions of all 4-mers in a 2-kb window surrounding the TSSs. After training, it separated 33,865 low-confidence clusters (not resembling known promoters) and 14,184 high-confidence clusters (resembling known promoters) (Fig. 2A,D). In some of the downstream analysis, we discard all low-confidence clusters, because they are less likely to represent full-length transcripts and thus do not have the functionality implied by their associated gene symbol and Gene Ontology (GO) terms (Ashburner et al. 2000).

### The translatome of Purkinje cells

**Figure 3.**
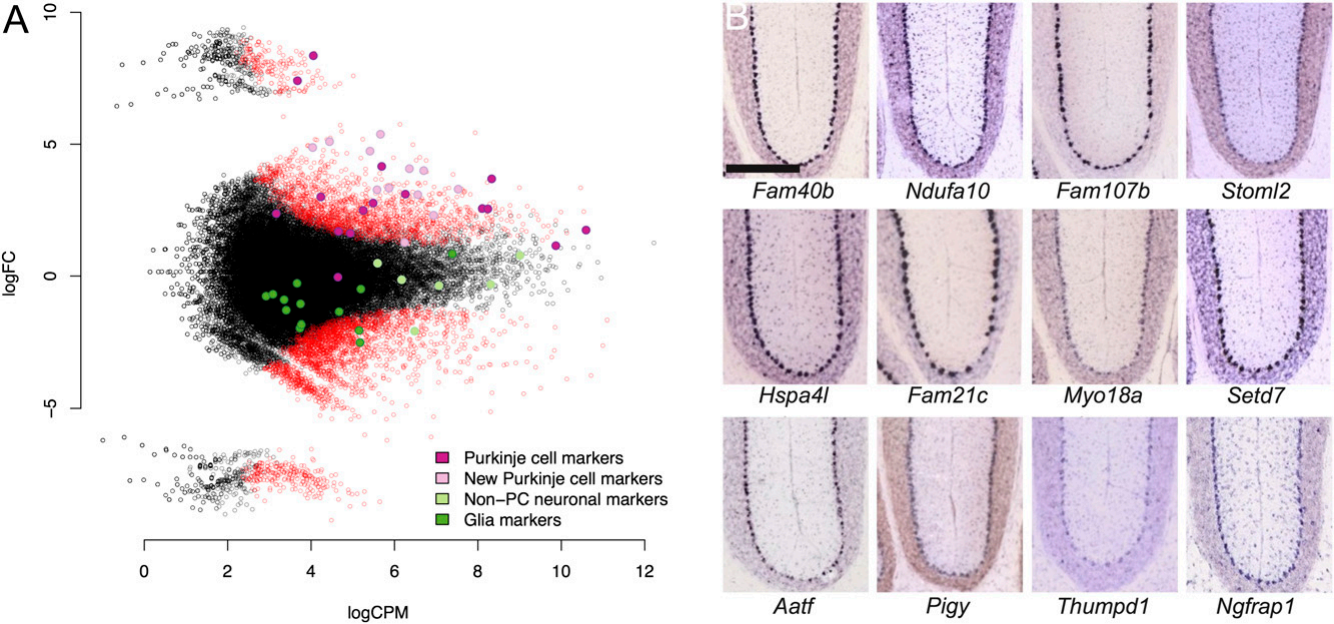
(*A*) Differential gene expression between the ribosome-captured (*up*) and control supernatant (*down*) samples. Each dot corresponds to a CAGEscan cluster. (*X*-axis) Gene expression level normalized by edgeR (log count per million); (*y*-axis) log_2_ fold change (FC) between the samples. The most extreme changes, where clusters group far from the main cloud of points, reflect a null expression in one of the samples. Open red symbols indicate clusters significantly enriched (positive FC)/depleted (negative FC) in PCs. Some clusters were annotated with markers for neuronal cells other than PCs, including granule cells (light green); glia markers (dark green); PC markers (dark magenta) and 12 clusters with strong enrichment in the bound fraction (light magenta), which can serve as novel PC markers. (*B*) Micrographs of sagittal sections showing in situ hybridization (Allen Brain Atlas) for the 12 new PC markers in mouse brain. Scale bar, 300 μm.

To quantify the relative transcript enrichment after ribosome capture, we compared the expression scores in the immunoprecipitated and control supernatant samples (Fig. 3A) using generalized linear models (GLMs) as implemented in edgeR (Robinson et al. 2010; McCarthy et al. 2012), and identified 1809 clusters significantly enriched (FDR ≤ 0.1), representing 16.8 ± 1% of the total expression count (for the top 25 enriched clusters, see Table 1; for the full list, see Supplemental Table S2 ). One hundred twenty-nine of these clusters did not have an Ensembl annotation. We could rescue 84 of them, for instance, where they would overlap with a RefSeq model or be in a long 3′ UTR (Miura et al. 2013) that is documented in human or mouse but not in rat transcript models. In total, we detected 866 different genes significantly enriched by the capture (Supplemental Table S3), including abundant transcripts that were missed by previous works (Fig. 3B).

**Table 1.**
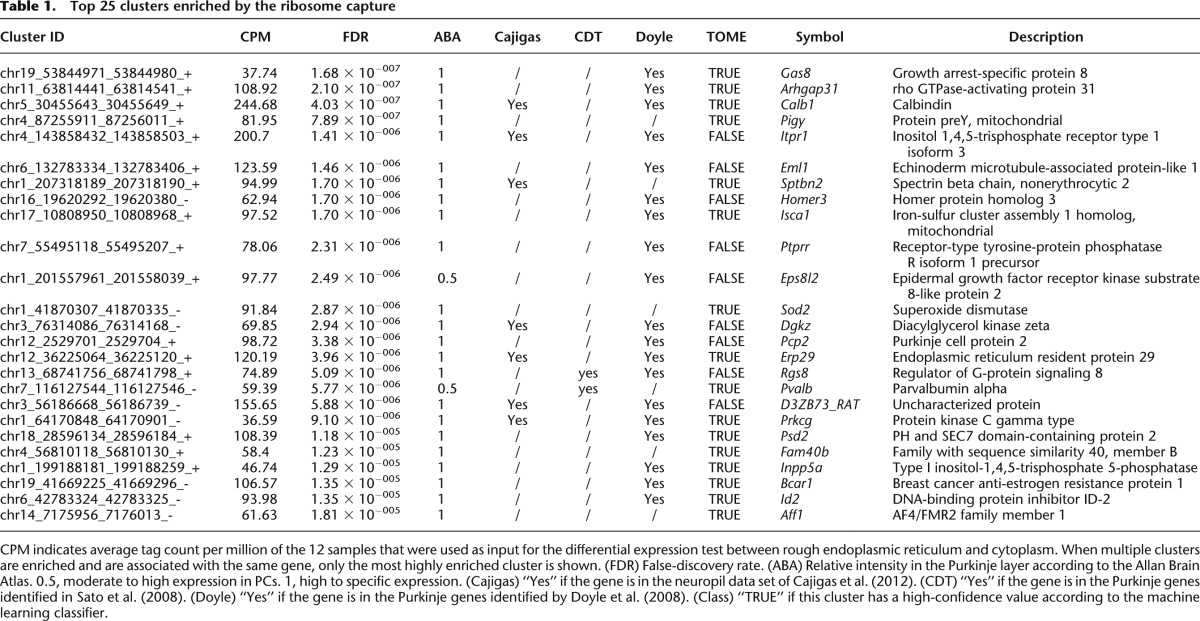
Top 25 clusters enriched by the ribosome capture

To confirm the selectivity of the enrichment, we examined marker genes selected through literature mining as representatives of cytoplasmic, plasma membrane, and ER membrane proteins. For each gene, we selected the most representative cluster, based on promoter classification and expression level. We then quantified the relative abundance of the markers for PCs (*Calb1*, *Dlg2*, *Pcp2*, *Pcp4*, *Itpr1*, *Lhx1*, *Ppp1r17*, *Car8*, *Grid2*, *Prkg1*, *Plcb4*, *Cacna1g*, *Homer3*, *Clmn*, *Gnaq*, *Rora*), as well as markers for glial cells (*Gfap*, *Slc1a3*, *S100b*, *Ppap2b*, *Fabp7*, *Sept4*) and neuronal cells other than PCs (*Calb2*, *Car4*, *Crtam*, *Reln*, *Grin2c*, *Grm4*, *Kcnd2*, *Chn2*, *Gprc5c*, *Serpini1*, *Pax6*, *Cacng2*, *Rbfox3*). The average log-fold changes for each of the three groups were 4.3 ± 2.3, −0.1 ± 2.8, and 0.7 ± 1.6, respectively, confirming the enrichment of Purkinje marker genes in the ribosome-captured libraries (Fig. 3A). Thus, while the PC population represents a fraction of the cells in the cerebellar cortex and were not all expressing the L10A-EYFP construct, we observed a specific enrichment of characterized PC marker genes, and at the same time a general depletion of marker genes of glial cells and neurons other than PC.

**Figure 4.**
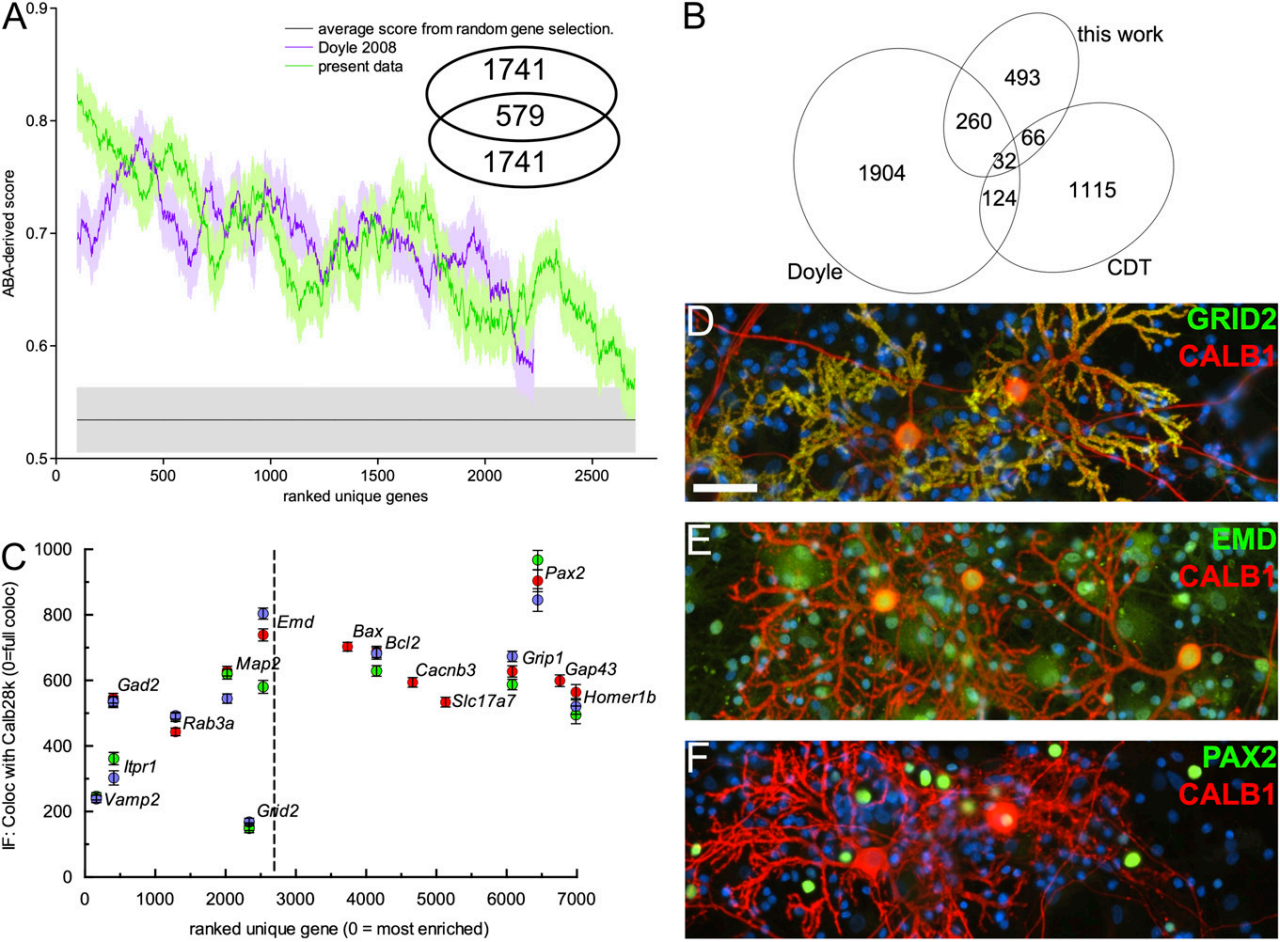
(*A*) Comparison of capture selectivity against a published data set and against noise. (*X*-axis) 200 points moving window (±SEM) averaging the ABA scores for our data set and that of Doyle et al. (2008), ranked by *P*-value. Noise was estimated from similar scoring of randomly selected rat genes. (*Inset*) Number of gene symbols unique or common to the 2320 first clusters plotted in this graph for each data set. (*B*) Venn diagram of the number of gene symbols unique or common to the sets of Purkinje-specific genes defined by this work, the experiment of Doyle et al. (2008) and the CDT-DB. The gene symbols can be found in Supplemental Table S4. (*C*) Comparison of transcript enrichment in PCs to the relative distribution of selected proteins, detected by immunofluoescence, in vitro. Anti-calbindin D28k (Alexa546) images were thresholded to define PC regions of interest. For all tested proteins, median fluorescence intensity in the non-PC area was expressed relative to intensity in PCs (with zero indicating exclusive expression by PCs). Measurements belonging to the same replicate are coded in the same color (red, green, blue). Note that *Grid2* (DELTA2R) appears here as an outlier because its most intense 5′ UTR cluster (rank, 129; LogCPM, 3.35) was not recognized as a promoter-binding region. (*D*–*F*) Representative micrographs for GRID2 (*D*), emerin (*E*), and PAX2 (*F*), all costained with anti-calbindin (red) and DAPI (blue). Scale bar, 50 µm.

A recent assessment (Okaty et al. 2011) indicated that TRAP applied to large brain regions suffers from higher contamination than methods using cell microdissection. Here we combined both approaches and evaluated the specificity of the capture by comparison with an orthogonal index of transcript expression, based on the in situ hybridization (ISH) micrographs of mouse cerebellum of the Allen Institute (Lein et al. 2007). Using microarrays, Doyle et al. (2008) identified 2320 known genes enriched in ribosome-captured RNA of PCs. We scored all the genes enriched by our ribosome capture (6590) and in the data set from Doyle et al. (2008) by inspecting the ISH staining pattern in the Purkinje layer relative to white matter, granular layer, and molecular layer, similar to the approach recently described by Dougherty et al. (2010). This Allen Brain Atlas–derived score (ABA score) for each gene was zero for ubiquitous or null expression, 0.5 for moderate to high relative intensity in the Purkinje layer, 1 for high to specific expression. When our data set and that of Doyle et al. (2008) were independently ranked according to FDR (false-discovery rate) and compared against the mean ABA score calculated over a sliding window (Fig. 4A), we observed the expected decreasing trend. Remarkably, although the Doyle et al. (2008) gene list and ours only partially overlap (Fig. 4A, inset, and 4B), the trend slopes are very similar. To evaluate this analysis against the null hypothesis (no enrichment), we randomly selected 700 genes from the Allen ISH data set and scored them as before. The ABA score for the first ∼2150 genes of the ranked Doyle data set and the first ∼2520 genes in ours are above the score of this random selection. Comparison with the ABA score, albeit imperfect because it indicates enrichment in the Purkinje layer rather than in PCs and thus also includes Bergman glia, suggests that microdissection and the use of nanoCAGE improves detection selectivity and sensitivity. This also suggests that the FDR threshold (0.1) chosen here is very conservative.

To assess the comprehensiveness of our search, we compared our list of enriched clusters to two related works in mouse (Fig. 4B). First, 292 out of the 2320 known genes enriched in PCs in the Doyle et al. (2008) data set were also significantly enriched in our libraries (33.7% overlap). Second, we also interrogated RIKEN’s Cerebellar Development Transcriptome Database (Sato et al. 2008), which reports 1337 genes expressed in PCs, of which 98 were enriched in our experiment (11.3% overlap). This cross-species validation further increases the confidence in the observed enrichment. The overlap between the three lists consists of 32 genes. Together with our finding of novel highly expressed markers (Fig. 3B), this suggests that none of the lists covers the PC-enriched translatome exhaustively. Extending our set of symbols to match the size of the Doyle et al. (2008) list by taking a FDR threshold of 0.5 still resulted in an overlap of <30% (Fig. 4A, inset). Thus, the 358 gene symbols common to our libraries and one of the two other data sets at the FDR threshold of 0.1 should be regarded as a high-confidence list of Purkinje-enriched mRNA. The clusters in each set of Figure 4B can be found in Supplemental Table S4.

To obtain an orthogonal evaluation of translating mRNAs enrichment in PCs and of its relevance to relative expression among cerebellar cortical cells, we quantified by immunofluorescence (IF) the presence of selected proteins in cerebellar primary cultures (Fig. 4C–F). Using calbindin staining to delineate PC and nuclei staining to assess local cell density, we computed the staining intensity of PCs for various proteins, relative to the staining of surrounding cells. The markers were selected based on availability of specific antibodies and to obtain samples evenly spaced along our ranked list of enriched transcripts. For transcripts within the top 2500 rank, we observed an approximate correlation between transcript rank and relative IF intensity (Fig. 4C). For transcripts above this rank (i.e., without any evidence of enrichment in PCs), the IF staining intensity was similar in PCs and non-PCs (Fig. 4C,E) for all tested proteins, except PAX2, which is selectively expressed by cerebellar interneurons (Fig. 4F). While the relative density of neuronal and glial populations may differ between cerebellum and cerebellar culture, the near-monolayer allowed for precise quantification with minimum staining background. Hence, these results confirm that the enrichment of translating RNAs measured in PCs is consistent with enrichment at the protein level.

### Motif search in promoters identifies a set of Purkinje-specific transcription factor binding sites

Next, we searched for transcription factors (TFs) regulating the PC translatome, by looking for binding sites overrepresented in the regulatory regions of promoters enriched in the ribosome capture compared with a background of cerebellar genes. Using the Clover algorithm (Frith et al. 2004), we identified 29 overrepresented motifs (Table 2; Supplemental File S2); of these, two belong to TFs required for normal cerebellar development (FOXC1 [Aldinger et al. 2009], ZFP423 [Warming et al. 2006]), while the RORA_2 motif is related to the RORA protein (isoform 2 in human) implicated in the function and maintenance of the PC layer (Boukhtouche et al. 2006). Conversely, we also identified 18 depleted motifs (Table 2). Among these, the depletion of the PAX6 motif is consistent with the high expression of the *Pax6* gene in the granular layer and with reports that it can also act as repressor (Duncan et al. 1998; Weasner et al. 2009). The enriched motifs had between 304 and 1569 potential targets (Table 2), suggesting that the transcriptome of PCs is regulated by large networks of genes.

**Table 2.**
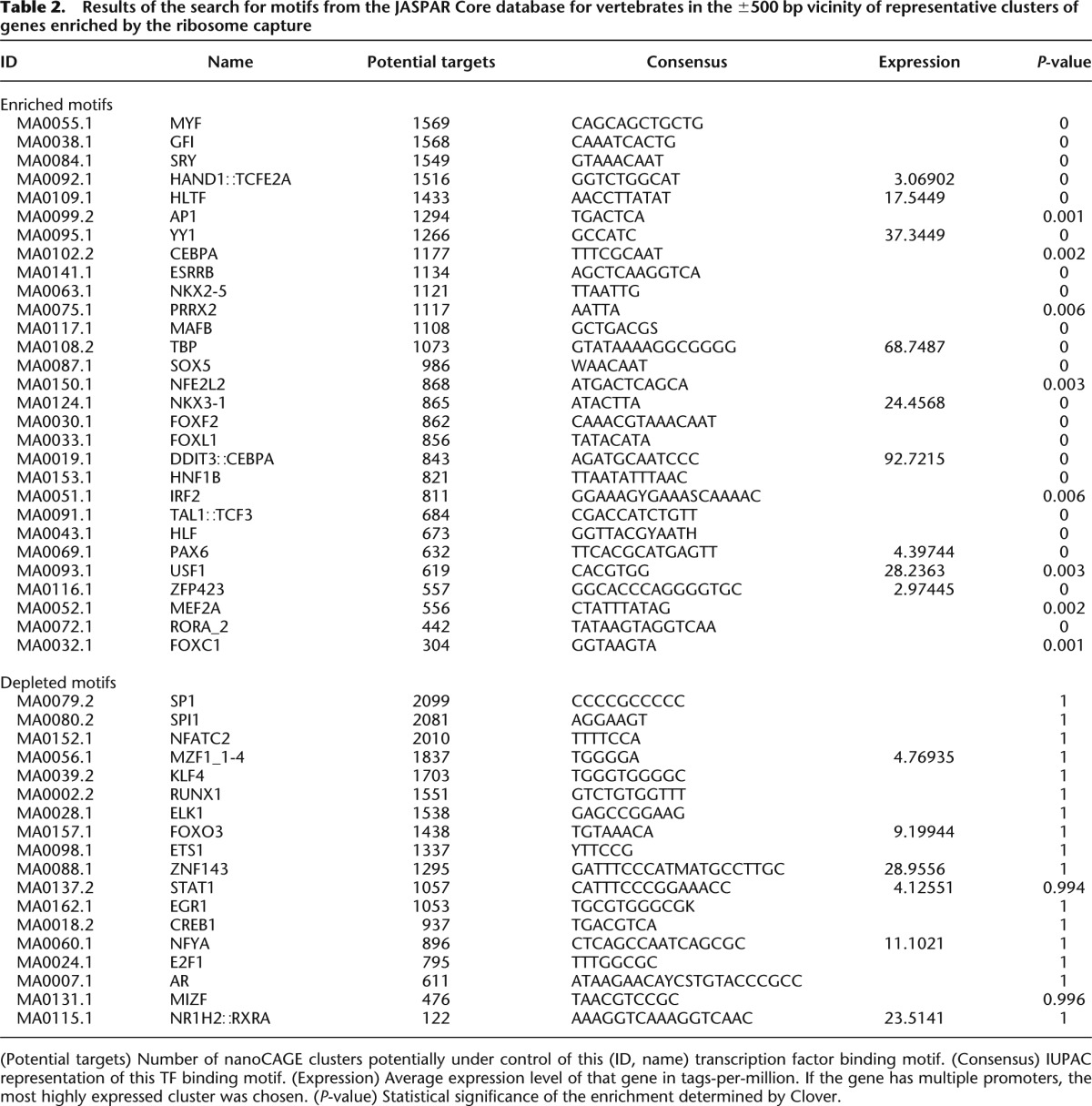
Results of the search for motifs from the JASPAR Core database for vertebrates in the ±500 bp vicinity of representative clusters of genes enriched by the ribosome capture

Motif search is restricted to predefined binding patterns. To identify entirely novel motifs that would be specifically enriched in PCs, we attempted a de novo motif discovery with AMD (Shi et al. 2011), which identified two short core motifs (GCGCGG and GCCGCG). However as these two motifs are very short and not very distinctive, we could not find a known TF binding site convincingly corresponding to any of them.

### The cytoplasmic and rER-bound translatome

We then investigated the difference between the cytoplasmic and rER-bound translatomes. In line with the different size profiles of the RNAs (Supplemental Fig. S2), the mean cDNA size was significantly smaller in the rER-bound ribosomes (288.8 vs. 724.8 bp; *P* = 5.0 × 10^−5^). We next compared how specialized the translatomes in the different compartments are, by calculating a richness score (Hurlbert 1971). It is defined as the mathematical expected value for the number of clusters to be observed if only 1000 tags per sample were distributed among them. High scores indicate that many clusters express similar numbers of tags, and low scores indicate that a few clusters are expressed much higher than the others. Because it is the expected value of a strong down-sampling, richness is very comparable across libraries that have very different sequencing depths, provided that they were prepared with the same method. The rER-bound translatomes were significantly richer than their cytoplasmic counterparts (908.6 and 893.5, respectively; *P* = 0.0065, paired *t*-test) (Supplemental Fig. S5A). This is consistent with the expression profile expected for a neuron, with a high diversity of membrane proteins involved in cell–cell interactions, ion homeostasis, and neurotransmission.

Ribosomes translating proteins addressed to the rER are first assembled in the cytoplasm and then bound by the signal recognition particle (SRP) before being transferred to the rER. As independent evidence that the isolated membrane fractions are enriched in ribosomes bound to the rER, we estimated the expression levels of the RNA component of the SRP ribonucleoprotein (SRP RNA; see Supplemental Methods) and observed a significant enrichment in this fraction compared with the cytoplasmic one (81.4 and 44.0 tags-per-million, respectively; *P* = 0.0004, paired *t*-test). Altogether, this shows that libraries from the membrane-containing fractions are enriched in transcripts bound to ER-associated ribosomes, either directly for translation or indirectly like the SRP RNA.

To identify mRNAs significantly overrepresented in either the rER or the cytoplasmic compartment, we compared six pairs of replicated libraries with GLMs, as previously. We detected, respectively, 9372 and 9950 clusters with an adjusted FDR-value ≤ 0.1 (Supplemental Fig. S5B; Supplemental Table S3), corresponding to 6565 unique Ensembl symbols. We then searched for Gene Ontology terms overrepresented in the 2201 gene symbols enriched in the rER, using the 7531 gene symbols of all other clusters as background. Using GOrilla (Eden et al. 2009), we identified terms in each GO domain (Supplemental Table S5), and used REVIGO (Supek et al. 2011) for clustering and visualization of the terms as a treemap (Supplemental Fig. S5C). Study of the cellular components confirmed the expected presence of transmembrane, luminal, or secreted proteins, with terms in branches of the ontology such as endoplasmic reticulum part, cation channel complex, neuron projection, and synapse part. Similar results were found with the biological process and molecular function domains (Supplemental Fig. S6; Supplemental Table S5).

### Biophysical translatome

**Figure 5.**
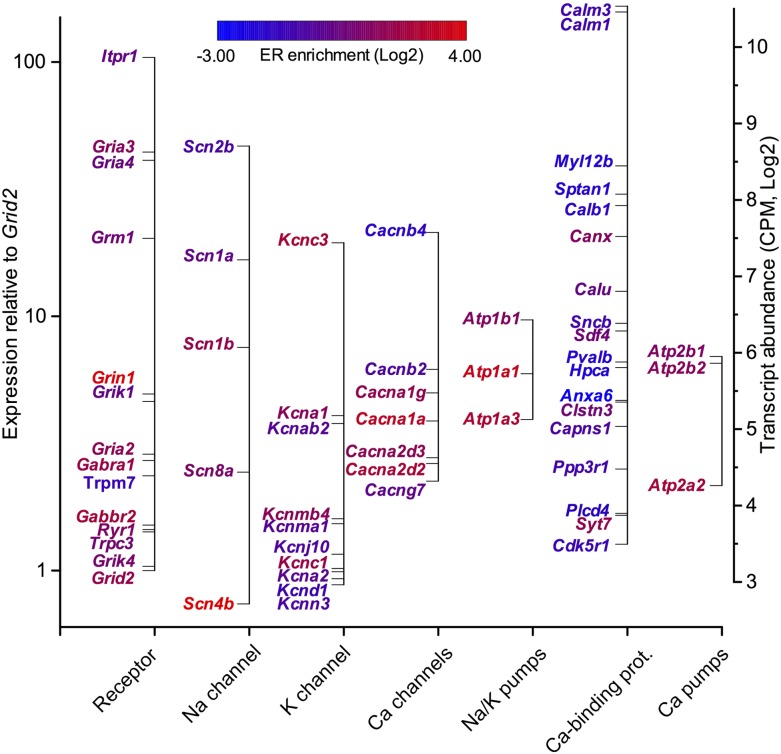
Relative expression of biophysically relevant transcripts coding for receptors, Na^+^, K^+^, and Ca^++^ channels, ion pumps, and Ca^++^-binding proteins. Expression levels are expressed relative to other transcripts in the library (*right* axis) and relative to the *Grid2* transcript coding for the PC synapse-specific glutamate receptor, ionotropic delta 2. Gene names are color-coded according to the differential expression between cytoplasm and ER fractions.

Our purification procedure gives access to a quantitative estimation of the relative transcript abundance in PCs and especially to the plasma-membrane proteins controlling the electrical properties of neurons. Accurate quantification of translating mRNAs may thus represent a proxy for measurement of protein synthesis. We identified 3068 clusters for 484 genes related to the control of PC membrane potential and ion homeostasis, including ionotropic and metabotropic synaptic transmission, ion channels, electrogenic ion transport, and calcium binding proteins. For detection robustness, we focused on the high-confidence, PCs-enriched (LogFC ≥ 0) clusters, for a total of 113 unique genes (Supplemental Table S6). Expression is represented as the relative abundance of each transcript in the libraries (Fig. 5, left-hand ordinate; Supplemental Table S3) and, for comparison with biophysical models, is represented relative to the amount of the *Grid2* transcript, among nine replicates (Fig. 5, right-hand ordinate). The *Grid2* product (glutamate receptor, ionotropic delta 2) is specifically expressed in PCs at the dendritic synapse and is thus relevant to describe the relative abundance of the transcripts related to electrical/synaptic activity.

Our biophysical translatome appears to densely sample all major ion channels and includes all the conductances previously used to build a biophysical model of PC, with realistic simulated electrical activity (De Schutter and Bower 1994; Miyasho et al. 2001). A comprehensive inspection of relative transcripts expression is described in the Supplemental Discussion. Interestingly, both for sodium and potassium channels, we observed a near stoichiometric ratio of the transcripts for the pore forming proteins and their respective regulatory subunits. This suggests that expression might be coregulated and also that the amount of translating transcript might be, for some proteins, a suitable proxy for relative protein level.

It should be noted that previous studies of the PC translatome (all based on the same data set) (Doyle et al. 2008; Heiman et al. 2008; Dougherty et al. 2010), only identified a fraction (∼28%) of these proteins, essentially the cytoplasmic beta subunits with a marked deficit for the pore-forming transmembrane subunits (∼11%). This suggests that separate isolation of ER-bound ribosomes is indispensable to detect the low-abundance, but highly relevant, transcripts of the ion channels, all highly enriched in the ER-bound fraction (Fig. 5, color-coded gene names).

In summary, the above analysis suggests that our approaches can be used to identify and quantify the full complement of the transcripts for proteins involved in the generation of PC’s electrical activity and integration of synaptic signals.

### The dendritic translatome

While numerous mRNAs have been identified in dendrites, only a handful have been verified to be translated locally under resting conditions, in the absence of plasticity-producing stimuli. To study long-distance RNA localization, we sequenced the cytoplasmic and rER-bound translatomes of the Purkinje dendrites themselves (Supplemental Table S3). EYFP-RPL10A expression in PC dendrites was barely detectable during dissection of live slices. This was in agreement with the weaker distribution of the endogenous RPL10A, as revealed by immunofluorescence compared to the strong somatic signal, as expected for the scattered distribution of dendritic polysomes (Supplemental Fig. S7; Spacek and Harris 1997).

We isolated the dendritic RNA of PCs by separating the Purkinje soma layer from the upper two-thirds of the molecular layer. RER can be found in the proximal dendrites of PCs, and polysomes are present even in the distal dendrites (Spacek and Harris 1997). The quantity of RNA harvested from >80 lobules was enough to prepare libraries with our standard protocol, albeit with a reduced precision on the expression values. We therefore focused on a qualitative exploration of the dendritic transcriptome. For additional immunity against contamination, a cluster (and its mRNA) was considered to be present in dendrites only if its expression from both the rER and cytoplasmic fractions is in the upper quartile of total expression from dendrites.

To characterize the dendritic transcripts, we searched for GO terms enriched in the dendritic clusters compared to all remaining clusters (Supplemental Table S7). Terms related to the mitochondria and energy production were among the most visible in all three subontologies, in particular cellular component (Supplemental Fig. S8). We also observed terms related to the synapse and its vesicles. In line with reports of local translation, we also found enrichment for terms related to ribosomal proteins and protein folding. More surprisingly, terms related to transport and cargo were also found, suggesting that the site of delivery takes an active part in this process.

To identify transcripts specifically enriched in dendrites relative to soma, we compared the dendritic ribosomes and each of the whole PC fractions using GLMs as previously, identifying 29 clusters (20 genes) with specific dendritic enrichment (Table 3). Consistent with a specific enrichment of translating mRNA from Purkinje dendrites, several of the identified mRNA are known to be locally translated in dendrites and to be highly enriched in PCs relative to other neurons in the cerebellar cortex (*Camk2a* [Ouyang et al. 1999], *Pcp2* [Wanner et al. 2000; Zhang et al. 2008], *Shank1* [Böckers et al. 2004]).

**Table 3.**
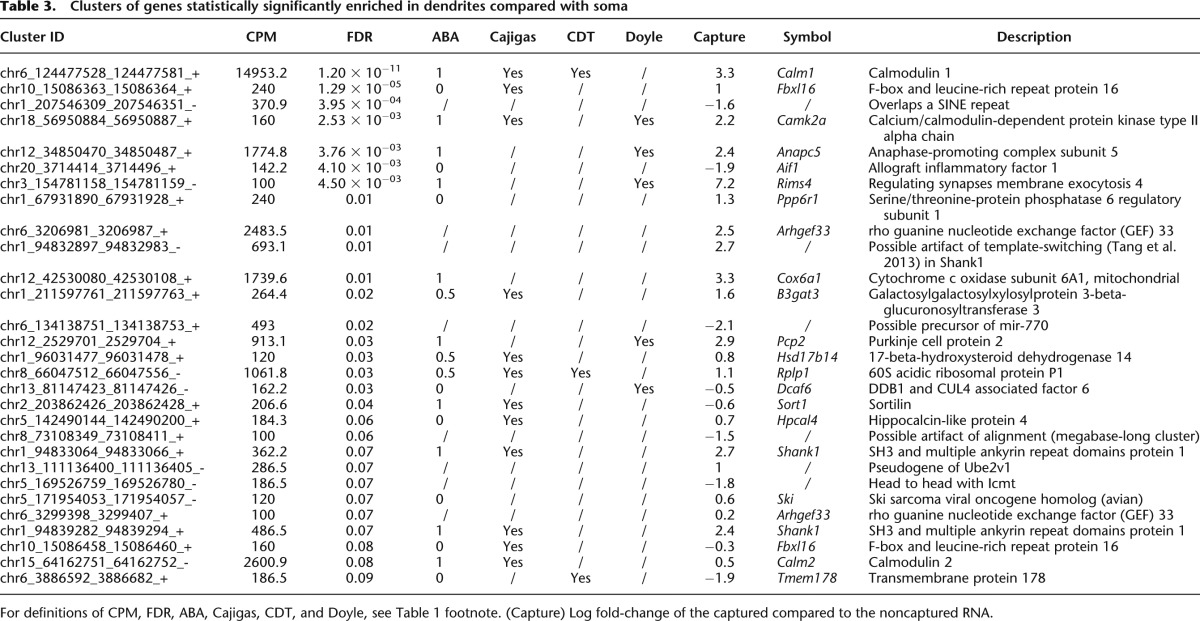
Clusters of genes statistically significantly enriched in dendrites compared with soma

While there have been several previous attempts to profile localized transcriptomes using a variety of experimental methods and model systems (Miyashiro et al. 1994; Moccia et al. 2003; Poon et al. 2006; Zhong et al. 2006), recently Cajigas et al. (2012) took advantage of the higher sensitivity of digital expression profiling to define the transcriptome in synaptic neuropil, where they report a set of 2550 genes being expressed. This set has an overlap of only 256 of the dendritic genes identified in our experiment (Table 3). Such a small overlap was to be expected, as the two data sets have very different characteristics: We sequenced only ribosome-bound transcripts, while Cajigas et al. (2012) sequenced the general transcriptome. Furthermore, two different technologies were used (RNA-seq and CAGEscan), and the experiments have been done on two different cell types in different brain regions. Therefore, the small set of gene symbols that do overlap between the two studies can be expected to identify genes that are an essential part of the biology of dendrites and are not specific to any particular neuronal type.

### The noncoding RNAs of the translatome

Noncoding RNAs (ncRNAs) such as *H19*, *Uchl1os*, or *Igf2as* have been reported to be bound to polysomes in other systems (Li et al. 1998; Carrieri et al. 2012; Duart-Garcia and Braunschweig 2013) and may modify translation of specific target mRNAs. To identify potential novel regulatory noncoding genes, we inspected the 45 significantly captured CAGEscan clusters that did not have an annotation. We discarded four as potential template-switching artifacts (Tang et al. 2013) and eight others that aligned in pseudogenes. We also excluded seven clusters that were hundreds of kilobases long and spanned multiple loci. Among the 15 clusters marked as “high-confidence” promoters by our classifier, some were overlapping with repeat elements, while others were uncharacterized transcripts head to head with *Cbln1*, *Lhx1*, *Oxsm*, all supported by conserved synteny and cross-aligned mouse cDNAs.

**Figure 6.**
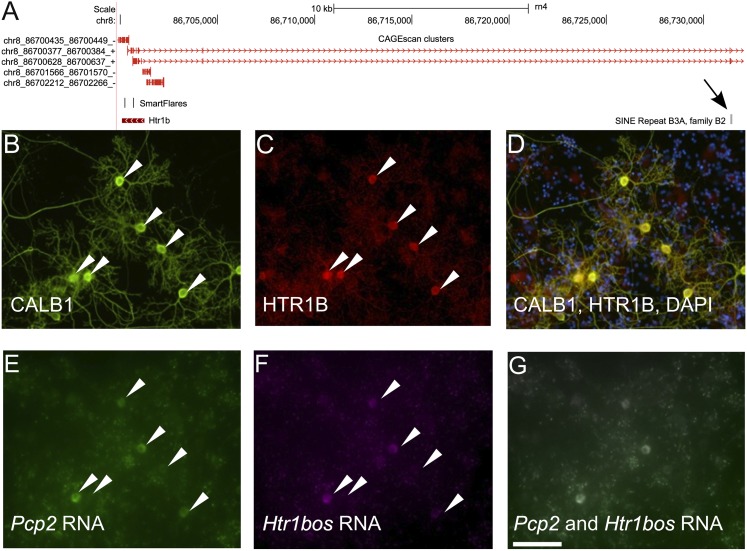
(*A*) Visualization of the *Htr1b* locus. (*B–D*) Immunofluorescence detection of CALB1 (*B*), HTR1B receptor (*C*), and combined immunofluorescence signals and DAPI staining (*D*). (*E*) SmartFlare *Pcp2*. (*F*) SmartFlare *Htr1bos*. (*G*) combined SmartFlare signals. Scale bar, 100 µm.

SINEUPs are a new class of functional long noncoding RNA, of which so far only two members have been identified (Carrieri et al. 2012). They overlap head to head with a protein-coding mRNA and, at the same time, with a SINE B2 repeat element downstream. This arrangement has been shown to increase protein-translation levels of *Uchl1* and *Uxt*, respectively, while not increasing the mRNA levels of these protein-encoding genes. Here, we found two candidate SINEUP RNAs, in *Htr1b* (5-hydroxytryptamine [serotonin] receptor 1B, G protein–coupled) (Fig. 6A) and *Srp72* (signal recognition particle 72). All *Htr1b* CAGEscan clusters were enriched in PCs and showed rER localization, in line with the transmembrane structure of the encoded protein. The potential SINEUP (which we term *Htr1bos* [5-hydroxytryptamine (serotonin) receptor 1B, G protein–coupled, opposite strand]) (S Laulederkind, pers. comm.) is found at higher levels in the cytoplasm compared with the rER, suggesting that the potential sense–antisense interaction may be dynamic.

We next validated the coexpression of both transcripts in live cerebellar primary culture (Fig. 6B–G). Transcripts for *Htr1b*, its SINEUP antisense, and the PC marker *Pcp2* were detected in live cells, using SmartFlares (Seferos et al. 2007): gold nanoparticles attached to oligonucleotidic probes duplexed to a fluorophore-conjugated strand, which are taken up into live cells by endocytosis. To confirm the presence of both the *Htr1b* transcript and the HTR1B protein specifically in PC, we used Cy3-coupled *Pcp2* probes together with Cy5-coupled probes for *Htr1b* mRNA. In addition, we combined SmartFlare detection with anti-HTR1B immunofluorescence with CALB1 as a PC-specific protein marker (Fig. 6B,E). The *Htr1b* staining was strongest in PCs, at both the protein (Fig. 6C) and RNA levels (Supplemental Fig. S9). Similarly, *Htr1bos* was only detected in PCs (Fig. 6F; Supplemental Fig. S9). Observation of the same cells in the live culture and after fixation/immunostaining allowed unambiguous identification of PCs and colocalization of the probes and markers (Fig. 6E,G). Forty-nine out of 49 PCP2^+^ PCs (from three culture batches) were found to be positive for *Htr1b* and 67/67 (two distinct 5′ UTR probes, from three batches) were positive for *Htr1bos*. This demonstrates that expression of the *Htr1b* mRNA/protein and the antisense transcript is restricted to PCs, and provides strong evidence of coexpression of the putative SINE ncRNA with its cogent target.

## Discussion

Here, we present an innovative approach to determine the translatome of a specific neuron. Targeting of a ribosome-capturing transgene to a rat Purkinje neuron by engineered AAV, coupled with microdissection and cellular fractionation, led to the identification of the translatomes of PC’s subcellular compartments (dendrites, cytoplasm, and rER). Combined with quantitative sequencing with the nanoCAGE and CAGEscan methods, we report a complete description of these defined compartments, which have a specific translational profile. In comparison with previous works using transgenics and microarrays, our study uncovered new markers and noncoding RNAs, demonstrating that our approach is fruitful even in systems that were previously screened. The use of AA viruses also opens the way to studies outside the short list of organisms where transgenesis is possible.

In the mouse BAC PCP2-EGFP-RPL10A transgenics previously used by Doyle et al. (2008), it has recently been estimated that ∼1% of the polyribosomes extracted from the cerebellum carry the tag (Darnell et al. 2011). This probably represents the amount of poly-ribosomes in Purkinje cells relative to the total number of ribosomes present in all the cells of the cerebellum and stresses the necessity for efficient PC-specific ribosome capture. The BAC transgenic used the weak *Pcp2* promoter, while we used post-natal virus-mediated expression of RPL10A-EYFP under a strong synthetic promoter (CAG). In practice both are PC specific and efficiently compete with the endogenous RPL10A for binding the ribosome complex. Our virus-based expression of the ribosome-capture probe, however, eliminates the dependency on transgenic mice (TRAP and RiboTag) and can be used in all species infected by AAV, including primates. While none of the known surface receptors for AAV2 and AAV8 (proteoglycan molecules, FGFR1, RPSA) (Summerford et al. 1999; Akache et al. 2006) are specifically expressed by PCs, we could obtain selective transduction of our EYFP-L10A probe by combining these serotypes. In the present context, this is fortunate as the expression of the transgene under a synthetic promoter is less likely to interfere with expression of endogenous transcripts.

Ribosome capture should be most efficient for polyribosomes since the immunoprecipitation simultaneously binds several ribosomes bound to the same mRNA. The polyribosome is typically a cytoplasmic structure, and we are not aware of a similar structure having been described for ER-bound ribosomes. In spite of this potential difficulty, our fractionation approach proved to be very efficient to analyze the translatome of rER-bound ribosomes, as shown by the massive enrichment for mRNAs encoding transmembrane proteins (Supplemental Fig. S5C).

The overlap with the neuropil transcriptome recently described by Cajigas et al. (2012) suggests that some transcripts are necessarily translated in dendrites, irrespective of the large difference in transcriptome observed for widely different neurons such as the CA1 pyramidal neuron and the PC. In addition to confirming the presence of specific transcripts in dendrites, their association with ribosomes adds evidence that they are locally translated and suggests that this dendritic synthesis is the norm for a much wider population of transcripts than previously thought. Considering that dendrites are ill-equipped in terms of specific organelles for maturation of proteins containing transmembrane domains, it was surprising that the dendritic translatome includes a large number of transcripts from the rER fraction, with many of them encoding secreted or membrane-spanning proteins. As observed in pyramidal neurons (Kacharmina et al. 2000; Horton and Ehlers 2003), the protein-synthesis competence of PC dendrites does not appear to be limited to soluble cytoplasmic proteins. This is correlated with the presence of N-glycosylation enzymes in distal regions of PC dendrites (Zanetta et al. 1983). We speculate that the positive bias toward rER is caused by a majority of dendritic polysomes being associated with a membranous structure, maybe even when translating soluble proteins.

Interestingly, comparison of our dendritic translatome with a recent analysis of PC synapse proteome indicated a large overlap (see Table S1 in Selimi et al. 2009). Out of 36 synaptic proteins identified by Selimi et al. (2009) with high confidence, we found that 21 transcripts were present in our dendritic translatome, suggesting that these synaptic proteins are locally synthesized. This notably includes proteins known for their critical involvement in synaptic regulation such as receptors (*Grid2*, *Gria2*, *Itpr1*), scaffolding protein (*Shank1*, *Grid2ip*, *Dlg2*), and plasticity-related signaling (*Camk2a*). In addition, we identified dendritic transcripts for several proteins (*Col18a1*, *Sptnb2*, *Actb*) that were considered likely to be contaminants in the proteomics-based study of Selimi et al. (2009). Since these transcripts are ribosome-bound and since it is unlikely that both a protein and its mRNA have nonspecific interaction during immunoprecipitation, we conclude that these transcripts and proteins are genuinely present in PC dendrites. This suggests that as much as two-thirds of synaptic proteins may be locally synthesized in dendrites. This supports previous conclusions based on the analysis of hippocampal neuropil (Cajigas et al. 2012) and synapse-associated transcripts in the forebrain (Suzuki et al. 2007). The successful parallel detection and quantification of dendritically translated mRNA with this approach opens new possibilities for large-scale detection of the protein synthesis associated with PC long-term synaptic plasticity (Linden 1996; Murashima and Hirano 1999; Karachot et al. 2001).

While most of the transcripts captured were expected to be protein-coding genes, the translatome consists of all RNA associated with ribosomes, and this includes functional noncoding RNA, such as the SRP RNA, and some instances of transcripts that could be functional ncRNA captured through antisense binding to their target. It is known that ncRNA generally have lower expression levels than mRNA (Djebali et al. 2012), so we expect that increasing the number of control supernatant libraries would allow the detection of more instances of possibly functional ncRNA in the translatome. We currently do not know whether the instances of ncRNA detected in our data set represent regulatory RNA with an exceptionally high copy number (i.e., the “tip of the iceberg”) or a small subclass of ribosome-associated ncRNAs. Further work would be needed to extend the coverage to capture small ncRNAs that were recently reported to be interacting with ribosomes in a regulatory manner (Gebetsberger et al. 2012; Zywicki et al. 2012); to our knowledge there is no whole-transcriptome method to quantify long and short RNAs at the same time.

The advances that we introduced here for cell-specific translatome study in nontransgenic animals cover different aspects that have been individually optimized for collection of ribosomes from specific subcellular neuronal compartments with distinct properties. Beyond the immediate interest for the description of Purkinje cells, we believe that the present approach can serve as a template for the study of other neurons in the central nervous system of rodents and primates.

## Methods

### Virus transfection

All procedures were approved by the RIKEN Ethics Committee on Animal Research (#H25-2-245). The EYFP-RPL10A construct was packaged into a mosaic AAV2/2-8 (Applied Viromics). Four-day-old rat pups received intracerebellar 10 μL injection of virus at 3 × 10^11^ gc/mL. Acute cerebellar slices (350 μm) were prepared 28–32 d later, and we microdissected lobules IV to IX to isolate the Purkinje layer and/or molecular layer. For each of the biological replicates, we pooled 50 to 64 lobules to eliminate influence of sex, batch, and lobule (for details, see Supplemental Methods).

### Ribosome capture and RNA extraction

Ribosome captures were mainly carried out as already described (Heiman et al. 2008) with modifications to extract separate fractions enriched in cytosolic ribosomes or ER-bound ribosomes by centrifugation (Fig. 1A), before solubilization in NP40 (1%) and DHPC (diheptanoyl-sn-phosphatidylcholine, 30 mM) detergent and immunoprecipitation using magnetic beads coated with rabbit polyclonal anti-GFP antibody (Abcam, ab290). RNA was extracted using the PureLink RNA micro kit (Invitrogen). For details of the procedure and reagents, see Supplemental Methods.

### CAGEscan libraries

The CAGEscan libraries were prepared as described by Salimullah et al. (2011) and Tang et al. (2013) using half of the recovered RNA. The multiplex indexes (“barcodes”) used for each library and their loading concentrations are indicated in Supplemental Table S1. The libraries NChi10050∼53 were outsourced to DNAFORM. The libraries were sequenced paired-end on HiSeq 2000 (Illumina) with a read length of 51 bases, demultiplexed, filtered, and aligned on the rn4 rat genome (Gibbs et al. 2004) paired-end using BWA version 0.5.9 (Li and Durbin 2009); for details, see Supplemental Methods. The CAGEscan 5′ mates were grouped in 48,049 clusters using the peak calling algorithm Paraclu (Frith et al. 2008) version 5 with default parameters. The 48,049 CAGEscan clusters seeded from these Paraclu clusters using the “CAGEscan-Clustering” software (http://fantom.gsc.riken.jp/software) can be found in Supplemental File S1. Each CAGEscan cluster was annotated with all gene symbols of Ensembl (downloaded March 19, 2013) that it intersects in sense orientation. The transcript classifier used on the CAGEscan clusters is available at http://tometools.sourceforge.net/.

### Statistical analysis

*T*-tests were calculated with the R Language and Environment for Statistical Computing, version 2.15.1 (The R Development Core Team 2003), and richness (Hurlbert 1971) was calculated using the Vegan R package (http://www.r-project.org/), version 2.0-3.

### SmartFlares

The presence of *Htr1b* mRNA and antisense in live cerebellar primary culture was tested using SmartFlares (Merck Millipore; original method published as “nano-flares” in Seferos et al. 2007). Probes were prepared for *Pcp2* (GGTTGAAGAAGCCTTCCTGGTCAGGTG), *Htr1b* (CTTCATCATCTCCCTGGTGATGCCTAT), and *Htr1bos* (AGCAGTCCAGCACCTCCTCCTCCGCTT and GCATCACCAGGGAGATGATGAAGAAGG), as well as scrambled control sequence. The probes were added to the culture medium for 6–10 h before live imaging in a phenol-red-free saline solution. After fixation in 4% paraformaldehyde, the cultures were processed for immunofluorescence detection of the Purkinje marker protein calbindin1 (mAb 300, Swant, SZ) and HTR1B (ab85937, Abcam). Both the SmartFlare images of live culture and the immunofluorescence data sets included phase-contrast images, used for coregistration of the images. Note that immunostaining after fixation and permeabilization revealed more PCs than initially assessed by *Pcp2* mRNA detection in live culture. Since both *Pcp2* and *Calb1* are robust PC markers and the high-density cerebellar culture is not mono-layer, this observation suggests that the intensity of the SmartFlare staining may be reduced when PCs are covered by other neurons and glia.

### Immunohistofluorescence quantification

Primary cerebellar cultures prepared from P19 embryonic primordium were fixed (4% paraformaldehyde, 9.25% sucrose) after 21–24 d in vitro. Triple-staining was performed overnight with anti-calbindin D28k (1/1000, mAb, Swant, SZ, Alexa594-conjugated secondary antibody, A21203) and antibodies (all 1/100, rabbit) against one of the target proteins (see Supplemental Table S8) and a donkey anti-rabbit Alexa488-conjugated secondary antibody (1/1000, A21206, Life Technologies), before counterstaining all nuclei with DAPI. Image analysis is detailed in the Supplemental Methods.

## Data access

The CAGEscan libraries were submitted to the DNA Data Bank of Japan Sequence Read Archive (DRA; http://trace.ddbj.nig.ac.jp/dra/index_e.html) under accession number DRA000893.
